# The regulatory landscape of retinoblastoma: a pathway analysis perspective

**DOI:** 10.1098/rsos.220031

**Published:** 2022-05-18

**Authors:** Laura Gómez-Romero, Diana E. Alvarez-Suarez, Enrique Hernández-Lemus, M. Verónica Ponce-Castañeda, Hugo Tovar

**Affiliations:** ^1^ Computational Genomics Division, National Institute of Genomic Medicine (INMEGEN), Mexico City, Mexico; ^2^ Medical Research Unit in Infectious Diseases, Hospital de Pediatría, CMN SXXI, Instituto Mexicano del Seguro Social, Mexico City, Mexico; ^3^ Pharmacology Department, CINVESTAV, Mexico City, Mexico; ^4^ Center for Complexity Sciences, National Autonomous University of Mexico (UNAM), Mexico City, Mexico

**Keywords:** retinoblastoma, functional enrichment analysis, master regulator analysis, expression microarrays, transcriptional regulation

## Abstract

Retinoblastoma (Rb) is a rare intraocular tumour in early childhood, with an approximate incidence of 1 in 18 000 live births. Experimental studies for Rb are complex due to the challenges associated with obtaining a normal retina to contrast with diseased tissue. In this work, we reanalyse a dataset that contains normal retina samples. We identified the individual genes whose expression is different in Rb in contrast with normal tissue, determined the pathways whose global expression pattern is more distant from the global expression observed in normal tissue, and finally, we identified which transcription factors regulate the highest number of differentially expressed genes (DEGs) and proposed as transcriptional master regulators (TMRs). The enrichment of DEGs in the phototransduction and retrograde endocannabinoid signalling pathways could be associated with abnormal behaviour of the processes leading to cellular differentiation and cellular proliferation. On the other hand, the TMRs nuclear receptor subfamily 5 group A member 2 and hepatocyte nuclear factor 4 gamma are involved in hepatocyte differentiation. Therefore, the enrichment of aberrant expression in these transcription factors could suggest an abnormal retina development that could be involved in Rb origin and progression.

## 1. Background

Retinoblastoma (Rb) is a rare intraocular malignant tumour from early childhood that originates in the retina with an incidence of 1 in 15 000 to 1 in 18 000 live births [1]. However, experimental studies for Rb are difficult due to the challenges associated with obtaining normal retina, the tissue on which this tumour originates, in addition to the rarity of the tumour and the age of the patients. Rb is considered the most robust clinical model of genetic predisposition to develop cancer. Epidemiological and molecular studies of the tumour led to the discovery of the first tumour suppressor gene, the Rb susceptibility gene *RB1* [2–5]. This tumour is a cornerstone of cancer research and has been widely studied with different molecular tools. It has been suggested that alterations in specific pathways across different genes result in cancer, so cancer has been proposed as a disease of biochemical pathways [6,7]. Identifying and quantifying altered pathways are essential for discovering possible therapeutic targets and understanding disease development [8–10]. Pathway analysis (PA) has been used to obtain functional insights with omic studies, especially in cancer-related research [11–14]. PA aims to analyse the data obtained via high-throughput technologies to detect relevant sets of genes altered in specific conditions. These approaches rely on the high-level organization of biological functions stored in structured databases (such as Kyoto Encyclopedia of Genes and Genomes (KEGG)) and the implementation of sophisticated statistical methods. Overrepresentation analysis (ORA), functional class scoring (FCS) and pathway topology-based analysis (PTB) are different approaches for PA [15,16].

ORA methods can detect statistically enriched biological processes from a list of differentially expressed genes (DEGs). Statistical significance will be reached if more DEGs belong to a specific pathway than expected by chance. By contrast, FCS methods overcome the limitation of focusing on a limited number of genes per pathway; these methods account for the expression level of all genes in a pathway or gene set to compute a metric that reflects the state of each pathway or gene set. Pathifier [10] is an FCS method that uses a principal curve approach to calculate the deregulation level for each pathway in each sample by measuring the sample's deviation from normal behaviour. This algorithm does not require detailed knowledge about the tested pathways or their interaction networks. This approach can be used to perform a functional mapping for every selected pathway on every given individual, an essential step towards personalized diagnostics and therapeutics. Additionally, in PTB methods, the pathway topology is integrated into the analysis.

A different approach, master regulators analysis (MRA), has been extensively used for the study of cancer [11–14,17–21]. MRA allows for the determination of key transcriptional regulators that could be responsible for the establishment of cellular and organismal phenotypes. It has been proposed that such master regulators control significant interactions, particularly those in regulatory bottlenecks [22]. MRA assumes that the more DEGs in tumour cells compared to healthy tissue are responsible for maintaining the tumour phenotype. Therefore, the analysis seeks transcription factors (TFs) that target DEGs. A transcriptional regulatory network is generated based on the statistical dependency between each TF expression pattern and the rest of the genes. The regulons per TF (the set of targets of a TF) are assembled, and the DEG content per regulon is assessed through gene set enrichment analysis (GSEA) [23]. The TF with a regulon (genes that are either induced or repressed directly or indirectly by the TF) with the highest normalized enrichment score (NES) will become a candidate transcriptional master regulator (TMR) [12,18]. Identifying the TMRs may facilitate the design of targeted interventions, another desirable instance towards personalized medicine.

In this work, we revisited the dataset generated and used by Kapatai *et al*. [24]. They were able to obtain samples from three normal retinas, and we think this dataset represents an invaluable opportunity to study Rb from a pathway-level perspective. We have done a pathway-level study in Rb. We obtained and analysed the DEGs from comparing the gene expression between Rb samples and the normal retina. Based on the DEGs, we enriched functional processes with an ORA analysis. We also calculated the deregulation score for each pathway with an FCS analysis by applying the Pathifier algorithm on the same dataset. By either identifying the pathways with a higher proportion of DEGs than expected by chance or identifying the pathways with a higher global expression difference compared to normal samples (higher deregulation score), we simplified the complexity of the model to a handful of relevant pathways. This dimensionality reduction makes the interpretation of the relevant pathways easier to manage, as they are thought to be directly associated with biological functions related to the Rb phenotype.

Furthermore, we also identified TF regulators performing an MRA, which we integrated into a pathway-level analysis by interrogating which TMRs control these pathways. We applied all these analyses to the Kapatai *et al*. dataset since the number of tumour samples and the presence of control samples offer a rare opportunity to study Rb with pathway-focused tools. In this study, we present a broader view of the transcriptomic context associated with the Rb phenotype by evaluating individual gene contributions, collective gene expression patterns via master regulators, and identifying functional consequences by deregulated pathways, thus presenting a clear landscape of the effects of gene deregulation underlying this pathologic phenotype.

## 2. Methods

### 2.1. Datasets

The Kapatai *et al*. [24] dataset with 20 samples of Rb was generated at Birmingham University and kindly provided by the authors. Normal tissue samples were obtained from the GEO database (https://www.ncbi.nlm.nih.gov/geo/): two normal adult retina (GSM607947, GSM607948) and one 96-day human fetal retina (GSM460264). All CEL files were built up through Affymetrix Human Gene 1.0 ST microarrays. Sample identifications match the original publication [24].

### 2.2. Data analysis

**Figure 1.**
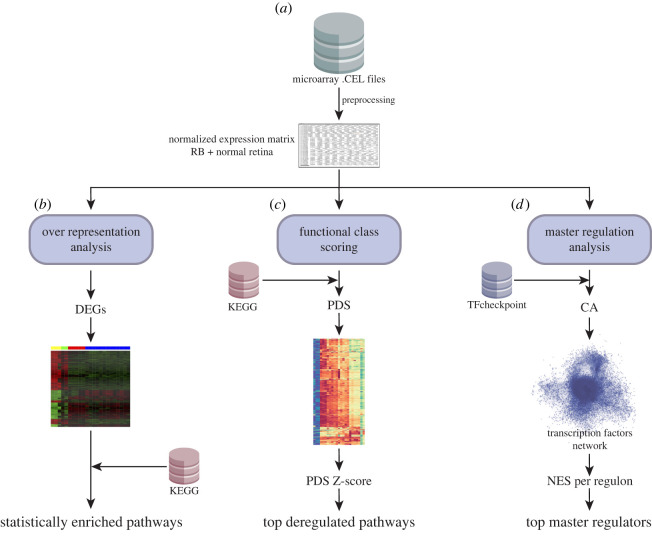
Workflow. (*a*) Raw data were pre-processed in the expression console software with the RMA algorithm to generate an expression matrix where each row is a gene, and each column is a sample. (*b*) ORA was performed to find the set of statistically enriched pathways after DEGs were found between Rb versus normal tissue. (*c*) Deregulation PA (a type of FCS) was performed using Pathifier. KEGG pathways were chosen as the reference database; a PDS and a PDS z-score were calculated per pathway to find the top most deregulated pathways. (*d*) Correlation analysis (CA) was performed to construct TF-gene regulatory networks using Correlation Tool (corto) and the human TFs from the TFCheckpoint database; only TFs with experimental evidence were included. The MRA algorithm used TF-regulatory networks and *t*-test-based differential expression to reveal TMRs.

The analysis pipeline was developed in three levels: differential expression, deregulation pathways analysis and master regulation analysis. (figure 1).

### 2.3. Pre-processing

The CEL files were pre-processed with the Affymetrix Expression Console software. The robust multichip average (RMA) algorithm was used to normalize and summarize probesets [25]. Pre-processed expression data were annotated with annotation files available in Affymetrix. The processing pipeline was performed in R Bioconductor [26,27].

### 2.4. Differential expression analysis and overrepresentation analysis

We calculated differential expression via the limma Bioconductor R package [28], contrasting normal retina versus Rb. DEGs (log-fold change (LFC) > 2 and adjusted *p*-value < 0.05) were grouped by hierarchical clustering (HC) (using Euclidean distance as distance method and complete agglomeration as clustering method) and the results plotted in a heatmap with gplots from R package [29]. Overrepresentation analysis was done using the Webgestalt tool [30]. We chose KEGG pathways like enrichment categories, Affymetrix Human Gene 1.0 ST IDs as the reference list and Benjamini and Hochberg as the multiple-testing correction method. We selected pathways with a false discovery rate (FDR) < 0.05 as statistically significant.

### 2.5. Functional class scoring analysis: pathway deregulation analysis

To determine the pathway deregulation score (PDS), we used the Pathifier algorithm [10], which uses the normalized gene expression matrix. Briefly, Pathifier applies the Hastie and Stueltzle algorithm [31] in a multi-dimensional space. Each axis represents a gene to calculate a principal curve that passes through and reaches all the points. Each point represents each gene's expression values that belong to one specific pathway in one sample. The centroid of the control group is the origin of the curve, and the distance of each point along the curve is the PDS for that sample–molecular pathway combination [10]. This process is reiterated, and a PDS is calculated for each sample and for each pathway considered. We used KEGG as a reference database of molecular pathways (296 metabolic pathways) [32]. The advantage of the KEGG database is that it is large, carefully curated and has integrated genomic and chemical information databases for biological processes [33]. Results for each sample and each pathway were plotted in a heatmap using Euclidean distance as distance metric, and Ward2 as the clustering method using gplots R Bioconductor package [29].

#### 2.5.1. Pathway deregulation score *z*-score

The PDSs obtained with Pathifier are based on comparing all samples through each single pathway. As different pathways may have different dynamic ranges, each PDS was *z*-score transformed to allow a fair comparison between different pathways. The mean and standard deviation of the PDSs per pathway were obtained and used to calculate the *z*-score per pathway per sample. This transformation allowed us to compare deregulation values across all pathways. Next, we obtained the median PDS *z*-score per pathway, taking into consideration either all tumoral samples or all samples per subgroup obtained with clustering analysis. From these, we can obtain the top 10 deregulated pathways in Rb or in each tumoral subgroup, respectively.

### 2.6. Master regulators analysis

We used corto (Correlation Tool) implemented in R [34] to generate a list of correlated TFs and target genes. For this analysis, we obtained the list of human TFs from the TFCheckpoint database [35]. We kept the TFs with experimental evidence that were found in the Human Gene 1.0ST microarray. Corto was run with 100 bootstraps, and interactions with a *p*-value less than 1 × 10^−7^ were chosen as true interactions [12]. This algorithm generates a regulatory network based on the correlation of expression, and a regulon is composed by all the target genes of a specific TF.

We used MRA, implemented in the package corto, to find the TMRs. MRA requires a regulatory network, a molecular signature and a null model. The network obtained by corto was used as the regulatory network. A *t*-statistic per gene was obtained from comparing the healthy versus control expression values, and it was transformed into *z*-scores. These *z*-scores were considered the molecular signature. Then, the gene labels were reshuffled, and this randomization was repeated 1000 times to obtain the null model. During the MRA, GSEA was run for each regulon using the *z*-scores as weights, and a NES was obtained. Each regulon NES was then compared with its null model distribution to calculate a *p*-value. The regulons with the highest NES value, a statistically significant *p*-value and with at least 20 genes were selected as TMRs [12,18]. Finally, we investigated the pathways regulated by each TMR.

## 3. Results

### 3.1. Differentially expressed genes are related to vision

Differential expression analysis allows us to measure quantitative changes in expression levels between two conditions. We found 305 DEGs (absolute LFC > 2) by comparing Rb samples to the normal retina, including 186 under-expressed genes and 119 over-expressed genes in Rb compared with the normal retina (see electronic supplementary material, table S1). Table 1 shows the first 10 genes most differentially expressed between both tissues, all of them with an absolute LFC ≥ 4.5 of them over-expressed (LFC ≥ 4) and 5 of them under-expressed (LFC ≤ −4).

**Table 1.** Top 10 DEGs. All with absolute LFC ≥ 4 and adjusted *p*-value < 1 × 10^−5^ between Rb and normal retina samples.

gene symbol	Ensembl	logFC	name	description
SNORD41	ENSG00000209702	5.47	small nucleolar RNA, C/D Box41	SNORD41 (small nucleolar RNA, C/D Box 41) is an RNA gene and is affiliated with the snoRNA class.^a^
SNORD115-23	ENSG00000201331	−4.65	small nucleolar RNA, C/D Box115-23	SNORD115-23 (small nucleolar RNA, C/D Box115-23) is an RNA Gene and is affiliated with the snoRNA class, a class of small non-coding RNAs associated with nucleotide chemical modification, such as methylation and pseudouridylation, by guiding and tethering partner enzymes to specific sites on RNA targets.^a^
SNORA71D	ENSG00000200354	4.59	small nucleolar RNA, H/ACA Box 71D	SNORA71D is an RNA Gene and is affiliated with the snoRNA class, a class of small non-coding RNAs associated with nucleotide chemical modification, such as methylation and pseudouridylation, by guiding and tethering partner enzymes to specific sites on RNA targets.^a^
SNORA23	ENSG00000201998	4.52	small nucleolar RNA, H/ACA Box 23	SNORA23 (small nucleolar RNA, H/ACA Box 23) is an RNA Gene and is affiliated with the snoRNA class.^a^ Has been found upregulated in human pancreatic ductal adenocarcinoma [36].
SNORA3A	ENSG00000200983	4.46	small nucleolar RNA, H/ACA Box 3A	SNORA3A (small nucleolar RNA, H/ACA Box 3A) is an RNA gene and is affiliated with the snoRNA class.^a^
CRABP1	ENSG00000166426	−4.03	cellular retinoic acid binding protein 1	this gene encodes a specific binding protein for a vitamin A family member and is thought to play an important role in retinoic acid-mediated differentiation and proliferation processes. It is structurally similar to the cellular retinol-binding proteins but binds only retinoic acid at specific sites within the nucleus, contributing to vitamin A-directed differentiation in epithelial tissue.^a^ CRABP1 has found that its under-expression in some other cancers such as breast cancer and ovarian adenocarcinoma is related to poor prognosis [37,38].
FABP7	ENSG00000164434	−4.30	fatty acid binding protein 7	the gene encodes a small, highly conserved cytoplasmic protein that binds long-chain fatty acids and other hydrophobic ligands. The encoded protein is essential in establishing the radial glial fibre in the developing brain. Alternative splicing and promoter usage result in multiple transcript variants encoding different isoforms.^a^
DPP4	ENSG00000197635	4.15	dipeptidyl peptidase 4	the DPP4 gene encodes dipeptidyl peptidase 4, which is identical to adenosine deaminase complexing protein-2, and the T-cell activation antigen CD26. It is an intrinsic type II transmembrane glycoprotein and a serine exopeptidase that cleaves X-proline dipeptides from the N-terminus of polypeptides. Dipeptidyl peptidase 4 is highly involved in glucose and insulin metabolism, as well as in immune regulation.^a^
SNORD115-25	ENSG00000199489	−4.03	small nucleolar RNA, C/D Box115-25	SNORD115-25 (small nucleolar RNA, C/D Box115-25) is an RNA Gene affiliated with the snoRNA class.^a^
NEFL	ENSG00000277586	−4.00	neurofilament light	neurofilaments are type IV intermediate filament heteropolymers composed of light, medium, and heavy chains. Neurofilaments comprise the exoskeleton, and they functionally maintain the neuronal calibre. They may also play a role in intracellular transport to axons and dendrites. This gene encodes the light chain neurofilament protein.^a^

^a^Information from www.genecards.org [39].

**Figure 2.**
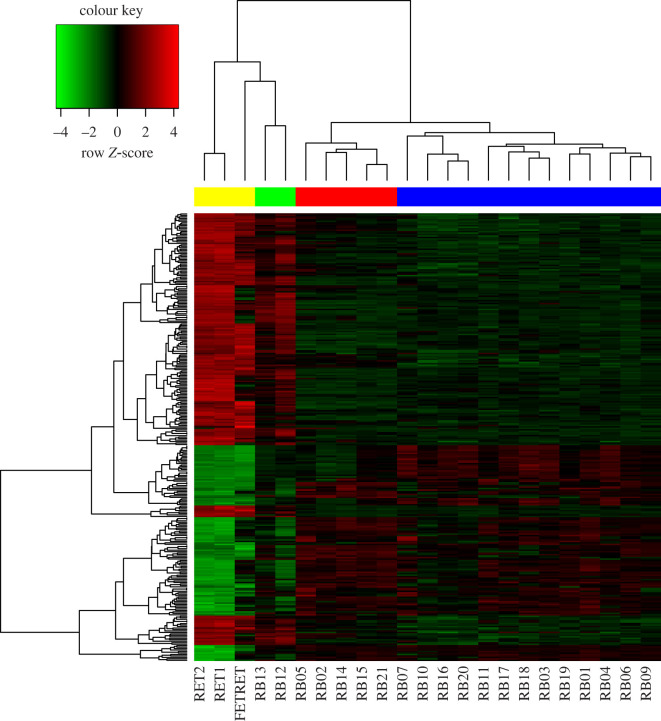
Heatmap of microarray expression *z*-scores computed for all genes that are differentially expressed (absolute LFC > 2 and adjusted *p*-value <0.05) between Rb (20 samples) and normal retina (3 samples). This heatmap shows the normalized expression values for 305 DEGs. Each row represents a gene and each column a sample (23 individual samples). Most Rb samples clustered in two branches indicated with the red and blue bars. Over-expressed genes are represented in red, and under-expressed genes are represented in green. Sample colour code: normal retina samples, yellow; Rb samples: green = normal-like, red = subgroup 2 and blue = subgroup 1. Normalization (z-score by row) was applied to improve visualization.

Unsupervised HC of DEGs showed four large clusters. As expected, this result exactly reproduces the grouping found by Kapatai *et al*. (figure 2). Cluster 1 is formed by 13 samples and corresponds to subgroup 1 described by Kapatai *et al*.; cluster 2 is composed of 5 samples and matches subgroup 2, also described by Kapatai *et al*. Interestingly, two tumoral samples are closely grouped with the control samples cluster (normal-like).

### 3.2. Overrepresentation analysis finds pathways related to cancer progression

We did an ORA based on KEGG pathways. The three pathways statistically enriched in DEGs with FDR < 0.05 were phototransduction (FDR = 3.30 × 10^−10^), olfactory transduction (FDR = 9.45 × 10^−6^) and retrograde endocannabinoid signalling (FDR = 0.04).

Both phototransduction and olfactory transduction are closely related metabolic pathways. Rhodopsin, the main protein responsible for converting light into electrical signals, is under-expressed with a LFC of −3.6975 (adj. *p*-value = 0.0059). Loss of this signal transduction capacity in Rb is related to the undifferentiated state these tumour cells exhibit and may explain the enrichment of these functions in DEGs. Additionally, some studies have found that components of this pathway, specifically members of the phosphodiesterase 6 (PDE6) family, may play a role in the development of breast cancer [36]. We found that PDE6A and PDE6G are also downregulated in Rb (LFC = −3.82 and −2.46, adj. *p*-value = 0.004 and 0.017, respectively). On the other hand, the olfactory transduction pathway is composed of a large number of genes from the G protein family. In recent years, the expression of these genes has been detected in multiple processes across different tissues [37,38,40–43] including some cancer-related functions.

For instance, some ORs (specifically OR2A4/7 and OR51B5) have been found to affect epidermal proliferation and differentiation [40]. Another study found that the activation of OR2J3 induces apoptosis and inhibits cell proliferation and migration in non-small-cell lung cancer cell lines [38]. Likewise, it has been found that some ORs (in particular OR2B6) are over-expressed in some types of cancer, such as breast and lung cancer [43]. Interestingly, OR2B6 is differentially over-expressed in our dataset. This suggests that the alteration of the expression of some family members could contribute to the establishment or permanence of cancer.

Lastly, changes in the expression pattern of the retrograde endocannabinoid signalling pathway (ECS) have been found in various cancer types, and such changes have been proposed to have an impact on disease progression and patient survival [44].

### 3.3. Pathway analysis reveals two retinoblastoma subgroups

We used the Pathifier algorithm [10] to determine pathway deregulation in Rb. Pathifier is an algorithm that combines the expression level of all genes within a single pathway or biological process, generating a unique deregulation value for each sample. This value is called the PDS. A PDS is calculated for each sample, and for each pathway, it reflects a deregulation value from 0 to 1. A value close to zero indicates no dysregulation with respect to normal tissue, and a value of 1 is assigned to the most deregulated sample or samples.

For each studied pathway, the tumoral samples with gene expression values similar to the average of control healthy tissue will have a PDS close to 0. By contrast, the samples with the highest PDS (close to 1) will have the most significant differences in gene expression levels compared to the average normal tissue. Previous studies have used this methodology to determine pathway deregulation of metabolic pathways in multiple cancer studies [45–49].

**Figure 3.**
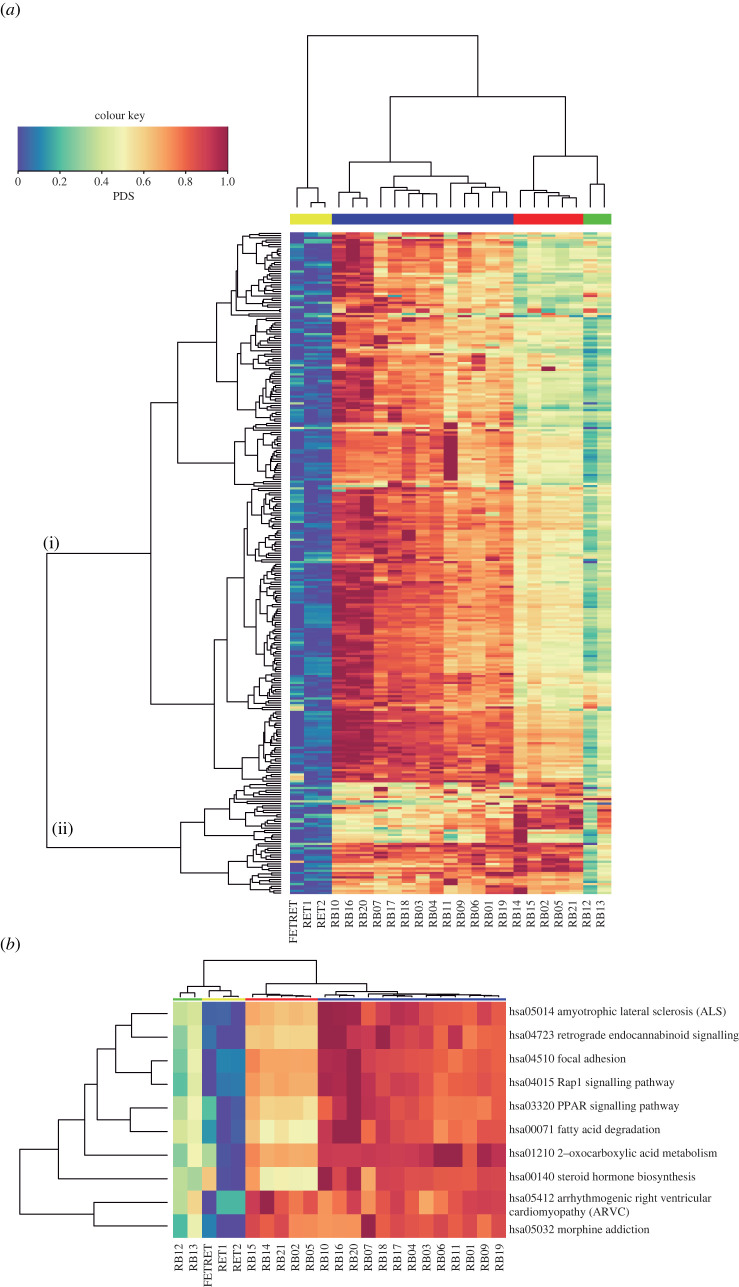
Deregulated pathways in Rb. (*a*) The heatmap indicates the PDS for each pathway for each Rb subgroup sample and normal retina sample. Rows represent 296 molecular pathways and columns correspond to individual samples. Pathways are grouped into two high-level clusters: a larger cluster composed of 246 pathways (i) and a small cluster composed of 50 pathways (ii). (*b*) The heatmap shows the top 10 most deregulated pathways; i.e. pathways with the highest median PDS z-score value. The column-colour key corresponds to sample colour code (same as in figure 1).

We estimated a PDS for each KEGG pathway (*N* = 296) for every sample. Unsupervised HC shows that deregulation levels are consistent with Rb subgroups. Figure 3*a* Illustrates that samples are grouped according to the same subgroups obtained previously via differential gene expression analysis (dendrogram at top subgroup 1, subgroup 2 and normal-like). However, the global clustering presents clear differences, grouping all Rb samples in a higher level cluster. On the other hand, pathways are clustered into two high-level clusters: a larger cluster composed of 246 pathways (cluster (i)) and a small cluster composed of 50 pathways (cluster (ii)). We can observe that pathways in cluster (i) tend to be more deregulated in subgroup 1 compared to subgroup 2 and normal-like samples. On the contrary, pathways in cluster (ii) tend to be more deregulated in subgroup 2 compared to subgroup 1 and normal-like samples. Also, we can see that most pathways tend to be less deregulated in normal-like samples compared to subgroup 1 and subgroup 2 samples; this could be caused by contamination with normal retina cells.

The most deregulated pathways in Rb were selected from the median PDS *z*-score calculation. The top 10 most deregulated pathways (i.e. with the highest median PDS *z*-score) were steroid hormone biosynthesis, focal adhesion, 2-oxocarboxylic acid metabolism, fatty acid degradation, Rap1 signalling pathway, peroxisome proliferator-activated receptor (PPAR) signalling pathway, morphine addiction, retrograde endocannabinoid signalling, amyotrophic lateral sclerosis (ALS) pathway and arrhythmogenic right ventricular cardiomyopathy (ARVC). These pathways were selected and grouped by unsupervised HC (figure 3*b*). This clustering shows that the normal-like samples are grouped with the normal retina and the two Rb subgroups are clustered together. Also, we can observe the same two high-level clusters of pathways as in the global deregulation heatmap: a top cluster that is more deregulated in subgroup 1 compared to subgroup 2 and the bottom one which seems to be equally deregulated in both subgroups (figure 3*b*).

We also determined which are the most deregulated pathways per Rb subgroup. Interestingly, the most deregulated pathways vary across subgroups (electronic supplementary material, table S2).

### 3.4. Master regulators analysis finds transcriptional master regulators involved in hepatocyte differentiation

We did an MRA to search possible TMRs in Rb. We used the corto algorithm to infer the regulatory network; this algorithm uses Spearman correlation supplemented with the data processing inequality between a list of previously defined TFs and genes to find statistical correlation from expression data [34,45,46]. This algorithm assumes that the most important regulators will be the ones affecting the expression of the highest number of their target genes in the condition of interest.

**Figure 4.**
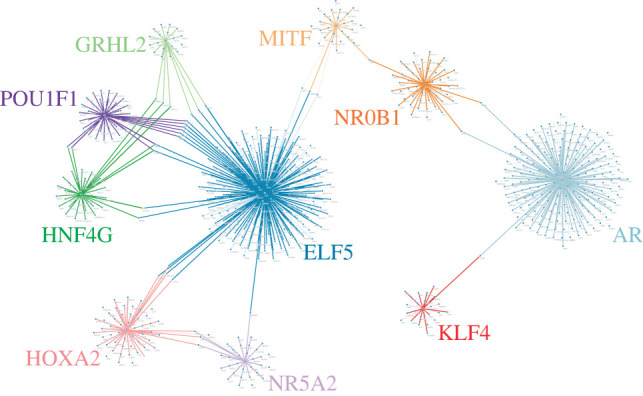
TMR networks. The ten most statistically significant TMR names are shown, and each colour indicates a TMR regulon. Each node represents a target gene, and each edge arriving to that node represents presumed regulation by a TMR.

Figure 4 shows the first 10 TMRs predicted by the MRA and their complete regulons. It can be noticed how the regulons vary widely in size and how there are few cases of coregulation of target genes by more than one TMR, with the notable exception of OR5AP2 and SLC22A10 that are regulated by three different TMRs (pituitary-specific positive transcription factor 1 (POU1F1), E74-like factor (ELF5) and hepatocyte nuclear factor 4 gamma (HNF4G)). The most statistically significant TMRs are presented in table 2 with a short description of their function.

**Table 2.** The most statistically significant TMRs and their functional description. NES = GSEA normalized enrichment score, *p*-value = NES *p* significance value, LFC = cases versus control TF LFC (* = adj. *p*-value < 0.05).

name	NES	*p*-value	LFC	function
NR5A2	6.43	1.25 × 10^−10^	1.06*	also known as liver receptor homolog-1 (LRH1), DNA binding zinc finger transcription factor, member of the fushi-tarazu factor 1
HNF4G	6.17	6.68 × 10^−10^	1.03*	a critical factor for hepatocyte differentiation
POU1F1	5.97	2.34 × 10^−9^	1.21*	a transcription factor for a growth hormone that regulates mammalian development
GRHL2	5.60	2.14 × 10^−8^	0.49*	it plays an important role in primary neurulation and in epithelial development [47,48]
MITF	5.58	2.45 × 10^−8^	0.36	it regulates melanocyte and optic cup derived retinal pigment epithelium development
HOXA2	5.34	9.09 × 10^−8^	1.68*	it may regulate gene expression, morphogenesis, and differentiation during embryonic development
KLF4	5.18	2.20 × 10^−7^	0.09	member of the SP1 family is required for normal development of skin barrier function. Remarkably this is one of the Yamanaka factors involved in the signalling network of pluripotency [49]
AR	5.00	5.80 × 10^−7^	0.16	it functions as a steroid hormone-activated transcription factor. Upon binding the hormone ligand, the receptor dissociates from accessory proteins, translocates into the nucleus, dimerizes, and then stimulates transcription of androgen responsive genes
ELF5	4.97	6.80 × 10^−7^	0.91*	it is a member of the ETS TF family, unique to animals, implicated in early development and cancer progression. It regulates epithelium specific genes and the later stages of terminal differentiation of keratinocytes
NR0B1	4.79	1.67 × 10^−6^	0.59*	it is a transcription factor that acts as a dominant-negative regulator of transcription which is mediated by the retinoic acid receptor

## 4. Discussion

In this work, we performed the functional analysis of Rb on three levels: differential gene expression, pathway deregulation and phenotype transcriptional regulation. Differential expression was carried out to characterize the gene level in contrast with the normal retina. We used Pathifier to analyse the biological functions and metabolic pathways level and MRA for the global transcriptional regulation level. In this approach, as a microscope focuses on different depths, we focus the analysis on different biological layers, and we find evidence of alterations at each level revealing the mechanisms behind Rb.

As previously mentioned, the main cluster features revealed by such analyses (figure 3*a*) are in agreement with previous findings by the group of Kapatai [24]. (i) Differential gene expression analysis and clustering resulted in two well-defined tumour subtypes plus a normal-like subset of samples that clustered together with the normal tissue. (ii) Overrepresentation analysis of differential genes using the KEGG database pointed out biological processes characteristic of retinal function with several downregulated genes and cancer progression related signalling. (iii) Pathway deregulation analysis uncovered both known and novel highly deregulated metabolic and signalling pathways. Interestingly, clustering analysis of the PDS by sample reveals two distinctive clusters with different sets of globally deregulated pathways. These two clusters correspond to a very high degree with the tumour subtypes first identified by Kapatai and co-workers and later confirmed by our differential expression analysis. This is relevant since this strongly suggests that the differences in gene expression profiles and deregulated pathways in both tumour subtypes are an actual consequence of distinctive regulatory programmes. (iv) In connection to this latter point, TMR analysis allowed us to find gene regulatory switches that may be behind such different transcriptional regulation programmes and, ultimately, perhaps behind phenotypic differences in the tumour subtypes.

Most of the DEGs found at this work (6 out of 10) belong to the snoRNA class, a small non-coding RNA class. One well-studied function of snoRNAs is their role in the modification, maturation and stabilization of rRNA. [50]. Emerging evidence has demonstrated significant roles of snoRNAs in cancer [51–53]. Mutations and aberrant expression of snoRNAs have been reported in cell transformation, tumorigenesis and metastasis, indicating that snoRNAs may serve as biomarkers and/or therapeutic targets of cancer [53]. Increasing evidence suggests that snoRNAs are closely associated with the TP53 regulatory pathway. For instance, a recent study identified that snoRNAs and fibrillarin (an enzymatic small nucleolar ribonucleoprotein) were usually over-expressed in human breast and prostate cancers, and this over-expression promoted tumorigenicity *in vitro* and *in vivo* [54].

Most top deregulated pathways have been previously associated with cancer. Steroid hormone biosynthesis is a pathway of recognized importance in the establishment and proliferation of various types of cancer [55–58]. Vertebrate steroid nuclear receptors (NRs) include the androgen receptor (AR), mineralocorticoid receptor, progesterone receptor, glucocorticoid receptor and estrogen receptor alpha [59]. There is very strong evidence of steroid hormone signalling acting as cancer drivers, at least in breast and prostate cancer and lymphoma [60–62]. Steroid hormones formed in a particular cell can activate the corresponding intracellular receptors of the NR superfamily, which act as TFs [56]. There is evidence that NR interactions with coactivators and corepressors are distorted in cancer, which ultimately disrupts the NR function [63–65].

On the other hand, focal adhesion plays an essential role in processes such as cell motility, cell proliferation, cell differentiation, regulation of gene expression and cell survival [66]. Closely related to focal adhesion is Rap1 signalling pathway. Ras-associated protein-1 (Rap1), a small GTPase in the Ras-related protein family, is an important regulator of essential cellular functions like formation and control of cell adhesions and junctions, cellular migration and polarization. Rap1 also regulates MAP kinase (MAPK) activity, playing many roles during cell invasion and metastasis in different cancers [67]. A common characteristic among tumour cells is their ability to reprogramme their metabolism [7,68–71]. The 2-oxocarboxylic acid metabolism pathway plays an important role in metabolic regulation [72–74]. Specifically, in cancer, 2-oxocarboxylic acid metabolism pathway seems to help cell proliferation indirectly through the mitochondrial electron transport chain via the metabolism of NAD^+^ [72,74]. The fatty acid degradation pathway is fundamental through the role that fatty acids play in cellular metabolism. Fatty acids are essential as structural components of the membrane matrix, secondary messengers and can also serve as fuel sources for energy in normal tissue. Lipid metabolism plays a fundamental role in processes such as metastasis, invasion or even tumorigenesis [75,76]. Moreover, particularly in cancer fatty acid, degradation could be related to the generation of substrates necessary for nucleotide biosynthesis rather than to energy generation [77].

PPARs are nuclear hormone receptors that are activated by fatty acids and their derivatives. PPAR protein family plays a role in processes like the clearance of circulating or cellular lipids via the regulation of gene expression involved in lipid metabolism in liver and skeletal muscle, lipid oxidation and cell proliferation [32]. It has been observed that the PPAR pathway can favour tumour progression through mechanisms such as induction of cell proliferation, inhibition of apoptosis, upregulation of VEGF expression, increased PGE2 production and COX expression [78], the latter responsible for the biosynthesis of prostanoids involved in mitosis and the inflammatory response [79].

Morphine addiction and retrograde endocannabinoid (ECS) signalling pathways are also closely related to carcinogenic processes [44]. Gamma-aminobutyric acid (GABA) is part of the morphine addiction pathway, and it is an important neurotransmitter for retinal neurons. It is involved in retinal maturation and development [80]. It has been shown that most Rb tumours are able to generate endogenous GABA via one or several different biosynthetic pathways [81]. Importantly, GABA is related to cancer cell metastasis and proliferation [82]. Retrograde ECS pathway is the pathway that intervenes in the regulation of endogenous cannabinoids that act as retrograde synaptic messengers in the brain. There is increasing evidence that the endocannabinoid system may be an important regulator of tumour cell malignancy [44,83,84]. However, there is contradictory evidence; it can stimulate tumour cell proliferation, angiogenesis and immunosuppression or inhibit tumour cell growth, angiogenesis, invasion and metastasis [84]. Noteworthy, some of these molecules have been recently proposed as possible therapeutic targets [44,83] since there is evidence that some non-psychoactive cannabinoids can have antimetastatic, anti-invasive effects [85,86] and antiproliferative capabilities [87] by inducing apoptosis [88] and autophagy [89,90].

The ALS pathway, which is a progressive and lethal motor disorder, includes in its gene list TP53, several members of the caspase family pathway, TNF and MAP kinases; all of these genes are widely documented as important agents in many cancer phenotypes [91–94]. Furthermore, a 2016 preclinical study showed how the C9orf72 knockout, the most common cause of ALS, caused an unexpected increase in tumours in mice [95]. On the other hand, more than half of the members of the ARVC pathway are known to be related to cancer, i.e. 20 (out of 77 members, 25%) integrins, 19 calcium voltage-gated channel-related proteins, TCF7 and two TCF7-like proteins [96–98].

Metabolic and signalling pathways previously implicated in Rb development were also found. Fatty acid synthesis is suggested to support an essential functional aspect of Rb. Most tumours present high levels of fatty acid synthase (FAS), and higher FAS expression has been correlated with more advanced choroid and optic nerve invasion, high mitotic index, and less differentiated histology [99].

Throughout the different sections of this work, we have presented the results of three different classes of pathway-level analysis derived from the same initial data: expression data from 29 Rb samples and three normal tumour-free retina samples. In table 3, we show the integration of our results. We present the pathways that are enriched in DEGs, the top 10 most deregulated pathways and the crosstalk between any of them and the regulons of each TMR. In what follows, we will discuss how the integration of these different levels of analysis led us to a unified view of the phenomena, from the molecular to the functional and up to the phenotypic levels.

**Table 3.** Pathways that are either enriched in DEGs (source = DE) or belong to the top 10 most deregulated pathways (source = PA) are listed. For each pathway, the enrichment ratio = number of DEGs that belong to the pathway/number of genes in the pathway (ER) and the FDR for the ORA are shown. Also, the ranking (RN) for the FCS analysis is shown. Finally, the number of genes regulated by any TMR is shown (N) along with the names and the number of genes regulated by each TMR.

source	description	DE		FCS	MRA										
		ER	FDR	RN	N	AR	ELF5	GRHL2	HNF4G	HOXA2	KLF4	MITF	NR0B1	NR5A2	POU1F1
DE	olfactory transduction	3.27	9.45 × 10^−6^	212	88	OR10Q1,OR11H6,OR7C2	OR10A4,OR10H3,OR10K2 (*N* = 32)	OR4A16,OR4B1,OR4D10 (*N* = 9)	OR2D3,OR4A16,OR51G2 (*N* = 18)	OR10AD1,OR10H2,OR10H3 (*N* = 13)	NA	OR8D2	OR10G7,OR1L1,OR7C2	OR10J1,OR10J5,OR10K1 (*N* = 10)	OR10G8,OR2F1,OR4F15 (*N* = 12)
DE	phototransduction	21.11	3.30 × 10^−10^	22	0	NA	NA	NA	NA	NA	NA	NA	NA	NA	NA
PA/DE	retrograde endocannabinoid signalling	3.68	0.040	8	1	NA	MAPK9	NA	NA	NA	NA	NA	NA	NA	NA
PA	amyotrophic lateral sclerosis (ALS)	5.27	0.16	4	10	C9orf72,DERL1,DNAH9	DCTN4,DNAH5,SETX,SPG11	NA	NA	COX4I1,UQCRC2	NA	NA	PPP3CA	COX4I1	NA
PA	fatty acid degradation	1.22	1	7	1	NA	ADH7	ADH7	NA	NA	NA	NA	NA	NA	NA
PA	steroid hormone biosynthesis	NA	NA	2	12	NA	UGT1A1,UGT1A10,UGT1A3 (*N* = 9)	NA	NA	HSD3B1	NA	NA	NA	NA	CYP3A7,UGT2B4
PA	2-oxocarboxylic acid metabolism	NA	NA	1	1	NA	NA	NA	NA	NA	NA	IDH3B	NA	NA	NA
PA	PPAR signalling pathway	1.47	1	9	0	NA	NA	NA	NA	NA	NA	NA	NA	NA	NA
PA	Rap1 signalling pathway	0.79	1	6	1	NA	FGF10	NA	NA	NA	NA	NA	NA	NA	NA
PA	focal adhesion	1.08	1	5	8	CHAD,ITGA11	COL6A6,LAMB4,MAPK9	NA	NA	NA	THBS2	NA	MYL2	LAMC2	NA
PA	morphine addiction	3.02	0.673	3	0	NA	NA	NA	NA	NA	NA	NA	NA	NA	NA
PA	arrhythmogenic right ventricular cardiomyopathy (ARVC)	2.24	1	10	2	ITGA11	NA	NA	NA	NA	NA	NA	NA	NA	CTNNA3

Only one pathway is enriched in DEGs and belongs to the top10 most deregulated pathways: retrograde endocannabinoid signalling. This pathway is also presumably regulated by one TMR: ELF5 regulates MAPK9. ELF5 is a transcription factor of the ETS family. This family is important for its role in cell proliferation, cell differentiation, cell development, apoptosis and tissue remodelling [100]. It has been observed that the deregulation of their members results in malignant cellular transformations [101]. Changes in the expression pattern of the genes in the endocannabinoid signalling pathway have been seen to impact disease progression and patient survival in various types of cancer [44].

The phototransduction pathway contains a lot more DEGs than the number expected by chance (highest enrichment ratio); besides, this pathway is one of the most highly deregulated (ranking 22). This fact is interesting, in particular since genes of the PDE6 family previously associated with the action of photoreceptors as signal transducers of luminal stimuli [102,103] have also recently been argued to be significantly over-expressed in human cancers, in particular in breast carcinomas [36]. PDE6A and PDE6G are present among the DEGs of this pathway. No gene in the phototransduction pathway is regulated by any of the proposed TMRs.

The olfactory transduction pathway has a noticeable behaviour as it is enriched in DEGs. It has a substantial number of genes regulated by the proposed TMRs (88 out of 427 total genes). This pathway is also significantly enriched (FDR < 0.05) for 6 of the 10 TMR regulons (ELF5, grainyhead-like transcription factor 2 (GRHL2), HNF4G, homeobox A2 transcription factor (HOXA2), nuclear receptor subfamily 5 group A member 2 (NR5A2) and POU1F1). This pathway comprises many molecules (448) involved in the transduction of chemical signals into the brain, including receptor proteins, olfactory specific Gs-proteins and type III adenylyl cyclases. Although the structure and function of its components in the nasal cavity are well known [104–106], there are still many unknowns about their role in other tissues [107]. However, they have been described as participating in secretion, migration, apoptosis, differentiation and cell growth [107]. Recent studies suggest the possibility of using some olfactory receptors as biomarkers of some types of cancer, such as OR51E1 and OR51E2 [107–111], which were found to be slightly over-expressed in Rb (LFC = 1.36 and 1.45 respectively, adjusted *p*-value < 0.001) and present in the HOXA2 and ELF5 regulons, respectively.

Although 2-oxocarboxylic acid metabolism is the most deregulated pathway, it is not enriched in DEGs, and it contains only one gene (IDH3B) regulated by a TMR: melanocyte-inducing transcription factor (MITF). Importantly, this interaction has been experimentally validated [112]. Furthermore, IDH3B is an important factor in the crosstalk between the cell cycle and the Krebs cycle [113]. Additionally, IDH3B has been shown to promote the transition between the G1 and S phase stimulating cell proliferation, and its over-expression has been related to poor survival [113]. Moreover, MITF is known to play an essential role during the differentiation of the retinal pigment epithelium and can regulate melanogenesis [114,115].

ALS is a highly deregulated pathway (ranking 4), and 10 of its genes are regulated by the proposed TMRs. Indeed, five different TMRs regulate ALS genes (AR, ELF5, HOXA2, nuclear receptor subfamily 0 group B member 1 (NR0B1) and NR5A2), several of which have been related to different types of cancer (C9orf72, multiple cancers; DERL1, non-small-cell lung cancer, bladder cancer, colon cancer, breast carcinoma; DNAH9, gastric adenocarcinoma; DCTN4, colon adenocarcinoma; COX4I1, skin cancer; UQCRC2, gastric cancer, glioma, testicular cancer, colorectal cancer, hepatocarcinoma; PPP3CA, ovarian cancer) [95,116–130]. Preclinical studies [95] showed how the C9orf72 knockout, the most common cause of ALS, caused the proliferation of unexpected tumours in different mouse tissues. It is important to highlight that C9orf72 appears as a target of the TMR AR in our regulatory network.

Hepatocyte differentiation was not dysregulated in our analysis but NR5A2 and HNF4G were identified as TMRs. NR5A2 is also known as hepatocytic TF, and HNF4G is a critical factor for hepatocyte differentiation. It has been reported that HNF4G can exert a carcinogenic effect by promoting cell proliferation and inhibiting cell apoptosis in lung cancer [131]. Moreover, NR5A2 transcriptional activation promotes pancreatic cancer progression [132].

There is, of course, a close relationship between Rb (the intrusive intraocular mostly pediatric tumour) and Rb (the tumour suppressor protein). It is widely known that Rb, when fully functional, often acts to prevent excessive cell growth by inhibiting cell cycle progression by binding to cell cycle proteins. Rb malfunction has been also linked to other cancers such as sarcomas, astrocytomas, melanomas, as well as other epithelial tumours [133], most noticeably breast [134], lung [135,136] and ovarian cancer [137]. This is unsurprising, since it has been argued that Rb is indeed a master regulator of the cell cycle, which is mutated or functionally inactivated in the majority of human cancers [138,139].

## 5. Conclusion

In this work, we studied Rb genetic expression at several organizational levels. For each of these levels, the existence of healthy tissue samples was of utmost importance. We identified the individual genes whose expression is different in Rb in contrast with normal tissue. We determined the pathways whose global expression pattern is more distant from the global expression observed in normal tissue. Finally, we identified which TFs regulated the highest number of DEGs and proposed them as TMRs. The integration of all these layers allows us to pinpoint genes and pathways relevant to the Rb phenotype.

Rb has been described as a highly undifferentiated malignancy. The enrichment of DEGs in the phototransduction and retrograde endocannabinoid signalling pathways could be associated with abnormal behaviour of the processes leading to cellular differentiation and cellular proliferation. On the other hand, the TMRs NR5A2 and HNF4G are involved in hepatocyte differentiation. The enrichment of aberrant expression in the regulons of these TFs could suggest an abnormal retina development which could be involved in Rb origin and progression.

## Data Availability

Publicly available datasets were analysed in this study. These data can be found in the Gene Expression Omnibus (GEO) database (National Center of Biotechnology and Information, NCBI), series GSE172170 (https://www.ncbi.nlm.nih.gov/geo/query/acc.cgi?acc=GSE172170), and the samples GSM460264 (https://www.ncbi.nlm.nih.gov/geo/query/acc.cgi?acc=GSM460264), GSM607947 (https://www.ncbi.nlm.nih.gov/geo/query/acc.cgi?acc=GSM607947) and GSM607948 (https://www.ncbi.nlm.nih.gov/geo/query/acc.cgi?acc=GSM607948). The electronic supplementary material, table S1, contains the list of 305 DEGs found in this work, with an adjusted *p*-value < 0.05 and an LFC > 2. The electronic supplementary material, table S2, lists the top 10 deregulated pathways per Rb subgroup. Median PDS z-score per pathway, taking into consideration all tumoral samples and all samples per subgroup [140].
